# Disappearance of Temporal Collinearity in Vertebrates and Its Eventual Reappearance

**DOI:** 10.3390/biology10101018

**Published:** 2021-10-09

**Authors:** Spyros Papageorgiou

**Affiliations:** Institute of Biosciences and Applications, NCSR ‘Demokritos’, 15310 Athens, Greece; spapage@bio.demokritos.gr

**Keywords:** HOX gene collinearity, spatial collinearity, temporal collinearity, TC disappearance, TC reappearence

## Abstract

**Simple Summary:**

In 1999 T. Kondo and D. Duboule performed excisions of posterior upstream DNA domains in mouse embryos and they observed that for an extended excision (including Evx gene) the Hox genes of the cluster were simultaneously expressed with the first Hoxd1 gene ‘as if’ Temporal Collinearity (TC) had disappeared. According to a Biophysical Model (BM) during Hox gene expression, Hox clusters behave similar toexpanding elastic springs. For the extended upstream DNA excision, BM predicts the TC disappearance and an experiment is proposed to test this BM prediction. In the chick limb bud C. Tickle et al. observed that the excision of the apical ectodermal ridge (AER) caused the inhibition of HoxA13 expression. However, the implantation of FGF soaked beads at the tip of the limb could surprisingly rescue HoxA13 expression after 24 h so that TC is restored.Brachyury transcription factor (TF) is essential in identifying the targets of this transcription and a chromatin immunoprecipitation microarray chip (ChIP-chip) was produced which can be inserted in the mouse embryonic cells. It is here proposed to insert this chip in the mutant cells where TC has disappeared and compare it to the limb bud case.Is TC restored? It is an important issue worth exploring.

**Abstract:**

It was observed that a cluster of ordered genes (Hox1, Hox2, Hox3…) in the genome are activated in the ontogenetic units (1, 2, 3 …) of an embryo along the Anterior/Posterior axis following the same order of the Hox genes. This Spatial Collinearity (SC) is very strange since it correlates events of very different spatial dimensions. It was later observed in vertebrates, that, in the above ordering, first is Hox1expressed in ontogenetic unit 1, followed later by Hox2 in unit 2 and even later Hox3 in unit 3. This temporal collinearity (TC) is an enigma and even to-day is explored in depth. In 1999 T. Kondo and D. Duboule, after posterior upstream extended DNA excisions, concluded that the Hox cluster behaves ‘as if’ TC disappears. Here the consideration of TC really disappearing is taken face value and its repercussions are analyzed. Furthermore, an experiment is proposed to test TC disappearance. An outcome of this experiment could be the reappearance (partial or total) of TC.

## 1. Introduction

Hox Gene Collinearity (HGC) is a fundamental property controlling development particularly in the early embryonic stages of vertebrates and many other animal phyla. E.B. Lewis was the first who observed this phenomenon in the *Drosophila BX-C* gene complex [1]. Lewis noticed that a class of genes (later denoted as Hox genes) are located in an ordered sequence (Hox1, Hox2, Hox3 …) along the direction 3′ to 5′ on the genome DNA. These genes are expressed in the same order in the embryo along the Anterior/Posterior axis. This common order in the chromosome and the embryo is called spatial collinearity (SC) [1,2]. SC is a strange property correlating entities at the macroscopic scale of the embryo (of the order of 1 mm) and the microscopic scale of the chromosome (of the order of 500 nm). This multiscale correlation (extending to about 4 orders of magnitude) is characteristic of Systems Biology [3]. Besides SC it was later observed, particularly in vertebrates, a temporal collinearity (TC). According to TC, the sequence (Hox1, Hox2, Hox3, …) are activated in a time order: first Hox1 is activated in ontogenetic unit 1, followed later by Hox2 in unit 2 and even later by Hox3,… [4].

In 1999 T. Kondo and D. Duboule (K-D) published a remarkable article describing several experiments performed in Duboule’s Laboratory [5]. K-D examined the regulatory region of vertebrate Hox gene clusters and in particular they investigated the posterior upstream DNA domain of the mouse HoxD cluster. They excised upstream domains of DNA of variable length up to an extremely large upstream domain cut-off (including Evx gene), and they tested the time of expression of Hoxd4 and Hod10 which lie anteriorly of the extended deleted region. K-D noticed that Hoxd4 and Hoxd10 are very prematurely expressed ‘at a time corresponding to that of Hoxd1 ‘as if’ temporal collinearity had disappeared’ [5] (I).

Here is presented a ‘prediction in retrospect’ of a Biophysical Model (BM) which predicts that TC disappeared indeed.

## 2. BM Formulation and Its Elastic Spring Approximation

**Figure 1:**
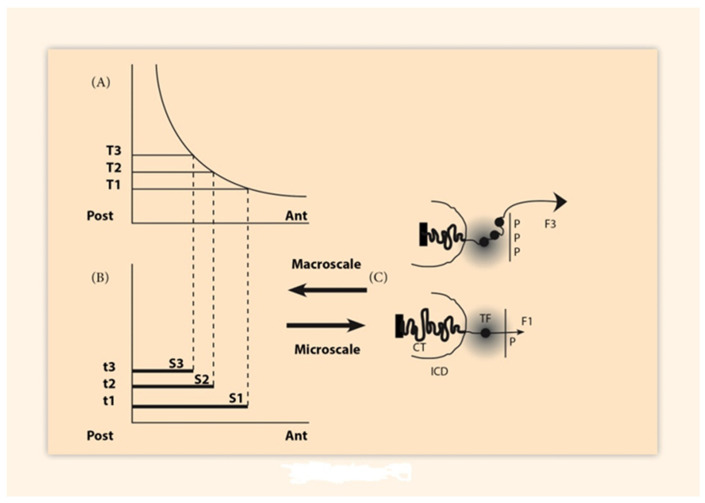
Morphogen gradient and Hox gene clustering. (Adapted from Y. Almirantis et al. Current Genomics, 2013, 14 (279–288). (**A**). Concentrations’ thresholds (T1, T2, T3) (**B**) Time sequences (t1, t2, t3) and corresponding domains (S1, S2, S3) determine the Hox1, Hox2, Hox3 activation in space and time. (**C**) (bottom) In an anterior cell of S1, a small force F1 pulls Hox1 (black spot) out of the chromatin territory (CT) toward the Interchromosome domain (ICD) and the regime of the Transcription Factory (TF) (grey domain). Allocation of polar molecule P opposite the telomeric end of the Hox cluster. At a later stage (top), in a more posterior location of S3, a stronger force F3 pulls Hox1, Hox2, Hox3 out of CT in the TF. (Allocation of 3P molecules).

The biophysical model (BM) was formulated after the K-D experiments. According to BM, physical forces are created at the telomeric end of the Hox cluster pulling sequentially the Hox genes towards a transcription factory domain where genes are transcribed [6,7] (Figure 1). At the same time technological advances made possible the measurement of geometricmodifications of Hox clusters during gene expressions. These modifications include cluster elongations along the 3′ to 5′ direction. Such elongations are naturally attributed to the pulling forces of the BM so that the activated Hox clusters behave similar to irreversibly expanding elastic springs [7].

**Figure 2:**
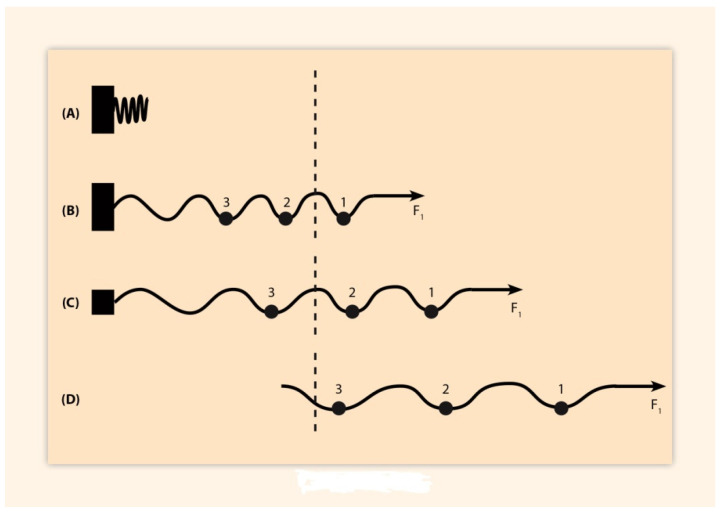
Elastic spring expansion (schematic). (Adapted from S. Papageorgiou J. Dev. Biol. 2021, 9(2) 17). (**A**) The compacted spring is at rest. (**B**) A small force F1 is applied to the right end of the spring. The spring fastening is complete (black orthogonal at the left end). The spring expands slightly and a small ball crosses the dashed line to the activation region. (**C**) The spring fastening is reduced (small black square at the left end). Two balls pass to the activation region. (**D**) The fastening is completely removed and, under the same force F1, all three balls are shifted into the activation region.

The proper function of an expanding spring depends not only on the pulling forces applied at one of the spring’s ends but also on the spring fastening at the other end of the spring. The former action is dynamic while the latter is static. The important role of both actions has been explicitly analyzed in [8]. Variations of the pulling forces, for a wide range of forces, are well described by Hooke’s empirical law: elongations are proportional to the measure of the pulling force. As for the role of cluster fastening, any force will slide and expand the spring in accordance with the degree of fastening of HoxA,D [8] (Figure 2). Therefore, both dynamic and static physical entities cooperate for the proper function of an elastic spring.

## 3. Spatial and Temporal Collinearities in the Vertebrates

### 3.1. Paradigm of the HoxA Expressions in the Chick Limb Bud

At this point it is constructive to examine another paradigm of Hox gene expressions after macroscopic manipulations at the embryonic level as performed in C. Tickle’s Laboratory [9] hereafter denoted as (**II**). In a particular experiment on chick limb buds, this team excised the apical ectodermal ridge (AER) of the bud (**II**). Then they examined the modified HoxA13 expression in the limb bud. The results are illuminating [9].

1. After the AER excision, HoxA13 is the first gene that rapidly switches off.
2. Upon continuous exposure of the limb bud to an FGF soaked bead, HoxA13 is rescued after at least 6 h.
3. HoxA13 is rescued depending on the dose of FGF soaked bead (the higher dose, the sooner rescue) [9,10].

**Figure 3:**
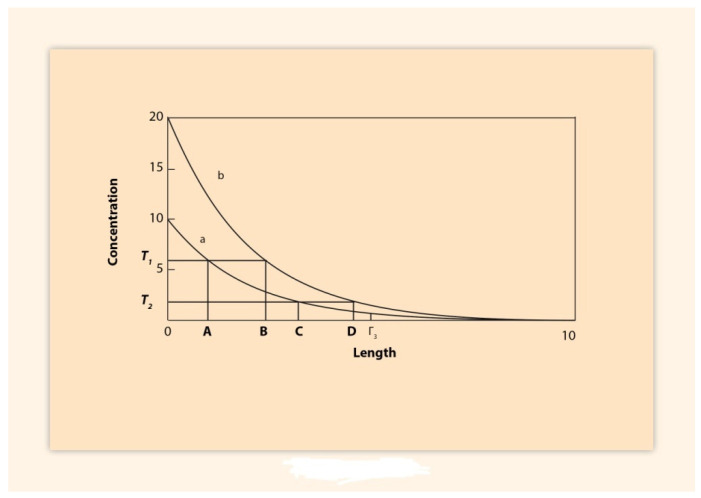
Variable diffusion gradients in arbitrary units of length and concentration. (Adapted from S. Papageorgiou, J Theor Biol.; 1998, 192: 43–53). At the origin **x = 0,** theconcentrations are **10** and **20** for the curves (**a**) and (**b**), respectively. For every point **x, b(x) = 2a(x).** This relation is true for any time t (0 ≤ t ≤ t (asymptotic).

In the chick limb bud long range action is mainly transmitted by passive diffusion of the morphogen which is produced at the distal end of the limb bud and spreads proximally. At the same time, the morphogen is degraded and finally a steady state morphogen distribution of decreasing exponential form is established with the concentration peak at the distal end [11] (Figure 3). In passive diffusion the velocity of signal propagation is not constant: at the start of diffusion, the spreading velocity is high whereas at later stages it gradually decreases [11]. In Figure 3 a morphogen gradient is depicted where the morphogen source varies. Further analysis is found in (**II**).

Tickle and collaborators removed the apical ectodermal ridge (AER) and noticed that after some hours HoxA13 switches off. However, if the FGF soaked beads are persistently inserted distally, the limb bud responds to this insertion and HoxA13 expression is later rescued. However, neither prematurely nor proximally extension of the expression is observed as would be expected according to the morphogen gradient model depicted in Figure 3 [11]. This indicates that the FGF gradient model is necessary but not sufficient for the HoxA expressions in the limb bud (**II**). Some other complementary mechanisms should be involved for the proper HoxA expressions [9,10].

The rationale in both paradigms **I** and **II** is the same: actions modifying Hox gene expressions are applied in Hox clusters and the resulting consequences are explored. (The common structure and ‘identity’ of the elastic spring and the Hox cluster is obvious). In Tickle’s Lab. the following (Exp. **II**) was performed in the chick limb.
**Exp II. ****    (a)**    
**→**   
**(b)**   
**→**     
**(c)**      
**→**     
**(d)**
(direct step)
** (af)**   
**←**   
**(b)**    
**←**    
**(c)**     
**←**     
**(d)**
(reverse step)

According to BM and its elastic spring approximation, state **(a)** represents the completely fastened spring without any force applied at the right end of the spring (Figure 2A). In (**Exp. II**) at state **(a)**, the AER is cut-off and substituted by a morphogen FGF bead. At state **(b)** a force **F** applies at the right end of the spring pulling this end of the spring beyond the dashed line (Figure 2B). In state **(c)** the spring fasteningis partly removed so that the spring is shifted further to the right (Figure 2C). In state **(d)** the spring fastening is completely removed and the spring is free to slide. Therefore, the force **F** pulls the whole spring beyond the dashed line (Figure 2D). The final state **(d)** is a mutant state which is the result of the AER substitution by the FGF bead.

In the reverse step, spring fastening is step by step restored by persistent application of the FGF bead: **(d)** **→(af).** Notethat the final state **(af)** differs from the starting state **(a)** since the morphogen gradient model is necessary but not sufficient for the HoxA expressions (see above). This indicates that the substitution of the AER by a morphogen FGF bead is only partly successful since supplementary mechanisms are needed for a complete substitution of the AER [9,10,11]. 

### 3.2. Paradigm of Gene Activation in the Mouse Embryo

The next step is to apply the rationale of paradigm **II** to paradigm **I** and compare the result. In the direct step of (**Exp. I**), state **(a)** of the mouse genome ends up to state **(d)** where a large posterior upstream DNA domain (including Evx) is cut-off so that, according to (**I**), TC has disappeared [5].
**(Exp. I) ****(a)**    
**→**   
**(b)**   
**→**     
**(c)**      
**→**    
**(d)**
(direct step)
**(a?)**   
**←**   
**(b?)** 
**←**    
**(c?)** 
**←**   
**(d)**
(reverse step)

It will be crucial to test whether the reverse step in (Exp. I) is feasible. This reverse step has not been performed as yet. It is necessary to complete this reverse step in order to properly compare **Exp.I** and **Exp.II.**

At this point a **diversion** is appropriate. For more than two decades, several groups have worked intensively in the field of TGF-beta signaling (involving Brachyury expression) in connection with other transcription factors such asFGF. In a recent review, it is recognized that Hox gene expressions are interconnected with the Brachyury regulatory gene expression [12]. One pioneering team, including JC Smith from Cambridge, recognized the important role of T-box Brachyury and showed that TGF-beta family signals cause gene spreading by a relay mechanism [13]. Furthermore, this team produced a chromatin immunoprecipitation microarray chip (ChIP-chip) which was applied to mouse embryonic stem cells to identify targets of Brachyury [14,15]. Independently, it was found that more than 500 target genes were identified in zebrafish [12]. These genes respond to signaling such asWnt or FGF [12]. The same signals are involved in both Exp.I and Exp.II. Based on this interconnection, it is a daring hypothesis to assume that the action of the FGF bead in Exp. II and the T-box XBRA action in Exp.I are comparable. To test this hypothesis the reverse step of Exp. I must be completed. 

Here it is proposed to apply the above novel microarray chip (ChIP-chip) and search for its effect in the reverse step of (**Exp. I**). In this reverse step, the mutant state **(d)** is the starting point where **TC disappears** according to (**I**). One cannot foresee what the result will be in the above search (see the following Section 4). 

## 4. Discussion

A. In the case of paradigm **I** the chip insertion operates in *terra incognita* and any prediction would be hazardous. Only Experiment can confirm a theory and fix the parameters involved in the data description. Note that an ideal Model is a ‘theory’ that predicts all experimental results in the realm of its implementation. Usually, a model can predict a limited set of results and all efforts aim at enlarging this domain by experimental trials. In the case of a microarray chip insertion the possible results one can anticipate are the following:
  - no response at all—no difference observed with or without pulling forces.
  - a partial restoration of HoxA13 expression **(b?, c?)**
  - a full rescue of HoxA13 **(a**?**)**

Only the experimental results will indicate the ‘correct’ answer.

B. A very important consequence of the above TC disappearance is its connection to TC loss as an evolutionary instrument applied to many animal phyla where TC is absent as, for instance, in *Drosophila* [8].
C. TC disappearance is a very striking event. A less striking effect is TC violation as observed by M. Kondo et al. in *Xenopus leavis* [16].

These authors analyzed the Allotetraploidizationsubgenomes during the Whole Genome Duplication and found that some of the two ‘homologs’ subgenomes (long and short L, S.) are not properly aligned in the Hox gene clusters as would be expected according to TC. They concluded therefore that TC may be violated.

## 5. Conclusions

1. A general observation could be that usually Experimentation precedes Theory and not *vice versa*: the French Flag was an experimental fact followed by the Positional Information Theory that described it [17]. However, in some rare cases this rule is not obeyed, as for example in the genius Turing Theory of Reaction-Diffusion in Morphogenesis. Turing predicted, without any preexisting evidence, that specific chemical reactions combined with Diffusion could spontaneously break the initially symmetric homogeneous state and lead to several inhomogeneous patterns as, e.g., the pattern formation in embryonic development [17]. This theory of Turing was later experimentally confirmed [18].

2. Some Hox gene expressions are positively or negatively regulated by upstream DNA regions beyond Hoxd13 [19]. For instance, Hoxb1 transgene relocated beyond Hoxd13 is strongly repressed in the fourth rhombomere (r4) whereas such a repression is not observed in the early mesoderm. It is usually observed that the expression of anterior Hox genes transposed posteriorly depends on the transposition location. Furthermore, the transcriptional stimulation of the relocated transgene depends on the time and the stimulation strength. Many attempts have been made to setup combinatorial rules reproducing the data. This approach depends on the specificities of time ant tissue location but a definitive formulation has not been obtained as yet [19].
